# Protected or Unprotected Fat Addition for Feedlot Lambs: Feeding Behavior, Carcass Traits, and Meat Quality

**DOI:** 10.3390/ani11020328

**Published:** 2021-01-28

**Authors:** Henry D. R. Alba, José E. de Freitas Júnior, Laudi C. Leite, José A. G. Azevêdo, Stefanie A. Santos, Douglas S. Pina, Luís G. A. Cirne, Carlindo S. Rodrigues, Willian P. Silva, Victor G. O. Lima, Manuela S. L. Tosto, Gleidson G. P. de Carvalho

**Affiliations:** 1 Department of Animal Science, Universidade Federal da Bahia, Salvador, 40170110 Bahia, Brazil; jose.esler@ufba.br (J.E.d.F.J.); stefanie_zootecnia@hotmail.com (S.A.S.); douglaspinaufba@gmail.com (D.S.P.); lgabrielcirne@hotmail.com (L.G.A.C.); carlindo.rodrigues@gmail.com (C.S.R.); willianzoo@yahoo.com.br (W.P.S.); victorgolima@hotmail.com (V.G.O.L.); manetosto@hotmail.com (M.S.L.T.); 2 Department of Animal Science, Universidade Federal do Recôncavo da Bahia, Cruz das Almas, 44380000 Bahia, Brazil; laudi.ufrb@gmail.com; 3 Department of Animal Science, Universidade Estadual de Santa Cruz, Ilhéus, 45662900 Bahia, Brazil; augustog@uesc.br

**Keywords:** calcium salt, fatty acid, lipid, ruminant nutrition, sheep

## Abstract

**Simple Summary:**

The use of lipids in ruminant diets aims to increase energy density without affecting the animal’s performance; however, its use can be toxic to the ruminal microbiota, which can be avoided with the use of protected fats. Diets with the inclusion of different fat sources (whole soybean grain, corn germ, calcium salt of fatty acids, and soybean oil) were tested to evaluate the effects of unprotected or protected fats on feeding behavior, carcass characteristics, and quality of the meat of feedlot lambs. The use of calcium salts from fatty acids in feedlot lambs’ diets improves the quantitative and qualitative characteristics of the carcass and meat.

**Abstract:**

This study aimed to evaluate the effect of the inclusion of protected or unprotected fats in the diet of feedlot lambs on feeding behavior, productive characteristics, carcass traits, and meat quality. Forty male Dorper × Santa Inês lambs (22.27 ± 2.79 kg) were randomly assigned to treatments in a completely randomized design. The experimental treatments consisted of five diets: no added fat (NAF), whole soybeans (WSB), calcium salts of fatty acids (CSFA), soybean oil (SO), and corn germ (CG). The total intake of dry matter (DMI) (*p* < 0.001) and neutral detergent fiber (NDFI) (*p* = 0.010) were higher in the CSFA and NAF diets. Feeding behavior, morphometric measurements, physicochemical characteristics, and centesimal composition of the *Longissimus lumborum* muscle were similar between treatments (*p* > 0.05). The CSFA diet provided higher production (*p* < 0.05) and better-quality carcasses. The inclusion of fat sources increased the concentration of polyunsaturated fatty acids (*p* < 0.05). The use of calcium salts of fatty acids in feedlot lambs’ diets provides better quantitative and qualitative characteristics of the meat and carcass.

## 1. Introduction

Increasing the energy density in ruminant diets is a practice that correlates with a higher proportion of available metabolizable energy (ME) and the consequent improvement in performance [1]. One method used for this purpose is the addition of lipids to the diet, which provide an energy density 2.25 times greater than carbohydrates [2].

According to the National Research Council (NRC) [3], exceeding the level of 7% of lipids in ruminant feeding can reduce dry matter intake due to the toxic effect on the ruminal microbiota. That occurs mainly if the fat source has a high content of unsaturated fatty acids (UFA) [4]. Although this toxicity mechanism has not been completely elucidated, the principal theory holds that this negative effect is related to the change in membrane fluidity in ruminal microorganisms [5].

Biohydrogenation is the mechanism by which ruminal bacteria protect themselves from UFA. Approximately 70–95% of linoleic acid and 85–100% of α-linolenic acid are biohydrogenated [6], producing saturated fatty acids (SFA), which will be absorbed in the intestine and directed to the final products, such as meat and milk [7]. 

The SFAs present in ruminant-derived products are a concern for human health since most of the population considers them responsible for the development of cardiovascular diseases, diabetes, obesity, and cancer [8]. With the use of protected lipids, which will not be biohydrogenated, it is possible to decrease the amount of SFA and to increase the UFA that reaches the small intestine [9].

Among the protected fats, we have those naturally protected, such as oilseeds, where the protein complex present in the cotyledon offers protection to lipids [10]. In chemically protected fats, the binding exerted between salt (main calcium) and fatty acids makes the latter resistant to metabolism of the microbiota of the ruminal environment [11].

Our hypothesis suggests that the addition of protected fat sources in the diet of feedlot lambs will improve feeding behavior and, consequently, the quantitative characteristics of the carcass and meat quality. This study was carried out to evaluate the effect of including fat sources, protected or not, on the feeding behavior, carcass traits, and meat quality of feedlot lambs.

## 2. Materials and Methods

### 2.1. Animals, Experimental Design, Management, and Diets

Forty non-castrated crossbred Dorper × Santa Inês male lambs (average body weight (BW) ± standard deviation: 22.27 ± 2.79 kg) at approximately 4 months of age were randomly assigned to treatments in a completely randomized design. Lambs were housed in individual covered pens (1 m^2^) with a suspended slatted floor, equipped with drinkers and feeding troughs.

Lambs were kept in a feedlot for 94 days, which were preceded by 15 days of adaptation dedicated to weighing, identifying, vaccinating, and treating the animals for endo- and ectoparasites. During this phase, animals received Tifton-85 hay as roughage (ad libitum) and increasing proportions of the experimental diets for adaptation using corn ground, soybean meal, soybean oil, whole soybean, calcium salts of fatty acids of soybean oil as the fat source (Megalac^®^-E, Vaccinar Ltd.a, Minas Gerais, Brazil), corn germ, urea, and mineral supplement specific for sheep as ingredients of the diets.

The experimental treatments (Table 1) consisted of five diets, corresponding to the use of four fat sources: (1) a basal diet without the addition of a fat source (NAF), (2) a diet containing whole soybean (WSB), (3) a diet containing calcium salts of fatty acids of soybean oil (CSFA, Megalac^®^-E, Vaccinar Ltd.a, Minas Gerais, Brazil), (4) a diet containing soybean oil (SO), and (5) a diet containing corn germ (CG). 

Diets were formulated as recommended by the NRC [12] to meet the nutritional requirements of lambs with an estimated weight gain of 200 g/day, with a roughage-to-concentrate ratio of 40:60. The feed was supplied twice daily, at 08.00 and 16.00 h. The supplied diets were weighed daily and adjusted to allow refusal of 10% of the dry matter supplied.

### 2.2. Sampling and Chemical Analyses

During the experimental period, samples were collected of the ingredients, diets, and refusals and were stored at −20 °C. At the end of the experiment, they were dried in a forced-air oven (55 °C for 72 h) and ground in a Wiley mill (model 0.48, Marconi, Piracicaba, Brazil) to pass through a 1-mm sieve.

Ingredients, diets, and refusals were analyzed according to the methods of the Association of Official Analytical Chemist (AOAC) [13] for dry matter (DM-method 930.15), crude protein (CP-Kjeldahl procedure; method 976.05), ether extract (EE-method 920.39), and ash (Ash-method 942.05). Neutral detergent fiber corrected to protein and ash (NDFpa), neutral detergent insoluble protein (NDIP), and acid detergent insoluble protein (ADIP) contents were estimated according to the method described by Van Soest et al. [14]. The contents of acid detergent fiber corrected to protein and ash (ADFap) and lignin were calculated as proposed by the AOAC (method 973.18) [15]. Non-fibrous carbohydrates (NFC) content was estimated according to Hall [16], and total digestible nutrients (TDN) were estimated according to the NRC [3] equations.

### 2.3. Feeding Behaviour

The amount of refusal feed was weighed every morning before feeding. Samples of the offered and refusal feed were collected for DM and NDF analysis. Total DM (DMI) and NDF (NDFI) intakes were measured for each animal based on the total feed offered and the refusal amount.

To register the time spent for feeding, rumination, and idling (h/day) visual observation of animals every 5 min for 24 h was used, performed by nine experienced observers (they were distributed into three groups which evaluated the feeding behavior for three consecutive hours per group), strategically placed so as not to disturb the animal’s comfort, starting at 08.00. The feeding and rumination efficiencies were obtained by dividing the average daily intake of DM and NDF with the total time spent in 24 h of feeding and rumination, respectively [17].

In three different periods of the day (10.00–12.00, 14.00–16.00, and 18.00–20.00 h), the number of chews and the time spent ruminating the bolus were recorded for each animal. To determine the number of daily cuds, the total rumination time was divided by the average time spent ruminating each cud [18].

### 2.4. Slaughter, Carcass Data, and Meat Samples

After solid fasting for 16.00 h, the animals were slaughtered following humanitarian procedures as required by the Brazilian legislation. First, the animals were numbed by stunning with a pneumatic gun in the atlanto-occipital region, followed by bleeding through the section of the carotid artery and jugular. Subsequently, skinning and evisceration were performed. Immediately, the hot carcass weight (HCW), pH, and temperature of the hot carcass were measured using a digital pHmeter equipped with penetrating electrode (HI-99163, Hanna^®^ instruments, São Paulo, Brazil) in the longissimus dorsi muscle between the 4th and 5th lumbar vertebras; following, the carcasses were stored in a cold chamber.

After 24 h of cooling at −4 °C, the carcasses were weighed to obtain the cold carcass weight (CCW) and the pH and temperature were measured. With those data, cooling losses (CL), cold carcass yield (Cold CY), and commercial carcass yield (Commercial CY) were estimated. On the same day, measurements for conformation, finishing, kidney fat, and morphometry were made [19].

The carcasses were sectioned, and the carcasses were divided into six commercial cuts (leg, loin, ribs, lower ribs, neck, and shoulder). In the right loin, a cross-sectional cut was made between the 12th and 13th ribs to expose the longissimus thoracis (LT) muscle to calculate the loin eye area (LEA), and according Cezar e Sousa [19], measurements for marbling and subcutaneous fat thickness in the *longissimus lumborum* (LL) muscle were made.

### 2.5. Physicochemical Meat Analysis

The meat color was evaluated using a Minolta CR-10 colorimeter (Konica^®^ Minolta, Osaka, Japan) that was previously calibrated with the CIELAB (Commission Internationale de l’Eclairage L, a*, b*) system using a blank tile, illuminant D65 and 10° as the standard observation. The LL muscle was sectioned and exposed to atmospheric air for 30 min before reading the oxygen myoglobin, immediately, the coordinates L* (lightness; L = 0 black, 100 white), a* (redness; ranges from green (−) to red (+)), and b* (yellowness; ranges from blue (−) to yellow (+)) were measured at three different points on the muscle [20].

According to the American Meat Science Association [21], to obtain cooking losses (CL), a LL muscle sample was cooked on a preheated grill (George Foreman Jumbo Grill GBZ6BW, Rio de Janeiro, Brazil) at 170 °C until the internal temperature of the steak center reached 71 °C, measured using a digital skewer thermometer (Salcasterm 200^®^, São Paulo, Brazil). The difference between the initial and final weights of a sample was used to determine CL. After cooling at room temperature, samples of approximately 2 × 1 × 1 cm were cut from the meat to be evaluated for Warner-Bratzler shear force (SF) using a TAXT2 texturometer (Stable Micro Systems Ltd., Vienna Court, UK) at 200 mm/min using standard shear blades (1.016 mm thick with a 3.05-mm blade) [22].

The centesimal composition of the LL meat samples was evaluated according to the methods of the AOAC [13] for dry matter (DM-method 930.15), crude protein (CP-Kjeldahl procedure; method 976.05), ether extract (EE-method 920.39), and ash (Ash-method 942.05).

### 2.6. Fatty Acid Profile

To obtain the fatty acid profile, LL meat samples employing the direct method of synthesis of fatty acid methyl esters (FAME) were used according O’Fallon et al. [23].

Fresh meat samples were homogenized by grinding them for 10–15 s in a coffee grinder at room temperature; immediately, the samples were dried by lyophilization for five days. The dried meat samples were made uniform by grinding them for 10–15 s in a coffee grinder at room temperature. Then, 0.5 g of a lyophilized sample was placed in a Pyrex culture tube with a screw cap (16 × 125 mm) to which 1.0 mL of the internal standard C19:0 (189-19 Sigma Aldrich, São Paulo, Brazil; 10 mg of C19:0/mL MeOH), 0.7 mL of 10N KOH, and 5.3 mL of MeOH were added.

The tube was incubated in a 55 °C water bath for 1.5 h with vigorous hand-shaking for 5 s every 20 min to properly permeate, dissolve, and hydrolyze the sample. After cooling below room temperature in a cold tap water bath, 0.58 mL of 24N H_2_SO_4_ was added. The tube was mixed by inversion and, with precipitated K_2_SO_4_ present, was incubated again in a 55 °C water bath for 1.5 h with hand-shaking for 5 s every 20 min. After FAME synthesis, the tube was cooled in a cold tap water bath. Three ml of hexane was added, and the tube was vortex-mixed for 5 min on a multi-tube vortex. The tube was centrifuged for 5 min in a tabletop centrifuge, and the hexane layer, containing the FAME, was placed into a gas chromatography (GC) vial. The vial was capped and placed at −20 °C until GC analysis.

The fatty acid composition of the FAME sample was determined by capillary GC on a SPTM-2560, 100 m × 25 mm × 0.2 µm of film thickness (Supelco), installed on a FOCUS GC gas chromatograph equipped with a flame ionization detector and split injection (Thermo Scientific Inc., São Paulo, Brazil). Hydrogenous (H_2_) was used as a carrier gas (1 mL/min), and nitrogen was used as an auxiliary gas. Detector and injector temperatures were set at 250 °C with a split ratio of 15:1. The oven temperature was set at 70 °C for 4 min, increased by 13 °C/min to 175 °C, held for 27 min, increased by 4 °C/min to 215 °C, and held for 31 min [24]. Fatty acids were identified by comparing their retention times with the fatty acid methyl standard described previously.

The atherogenicity (AI) and thrombogenicity (TI) indexes [25] and the hypocholesterolemic–hypercholesterolemic (h:H) fatty acid ratio [26] were calculated according to the following equations:

AI = [(C12:0 + (4 × C14:0) + C16:0)]/(∑ AGMI + ∑ω6 + ∑ω3)

TI = (C14:0 + C16:0 + C18:0)/[(0.5×∑ AGMI) + (0.5×∑ω6+ (3×∑ω3) + (∑ω3/∑ω6)])

h:H = (C18:1 *cis9* + C18:2 ω6 + 20:4 ω6 + C18:3 ω3 + C20:5 ω3 + C22:5 ω3 + C22:6 ω3)/(C14:0 + C16:0)

Desirable fatty acids (DFA) were obtained according to Rhee [27]. The activity indexes of the elongase and Δ^9^-desaturase enzymes were determined using the methodology proposed by Malau-Aduli et al. [28] and Kazala et al. [29], respectively.

### 2.7. Statistical Analyses

Data were subjected to analysis of variance (ANOVA) in a completely randomized design. Mean comparisons were done by Tukey’s test, with statistical probability of up to 5% using the PROC MIXED of SAS 9.4 software, according to the following model:

Ŷij = μ + Ti + εij

where Ŷij is the observed value of the dependent variable, μ is the overall mean, Ti is the fixed effect of the fat source, and εij is the experimental random error associated with each presupposition observation NID~(0, σ2).

## 3. Results

### 3.1. Feeding Behaviour

There was a higher DMI (*p* < 0.001) and NDFI (*p* = 0.010) in animals fed NAF and CSFA diets (Table 2). The rumination, feeding, idling, and chewing times and the DM and NDF feeding efficiencies of feedlot lambs were not influenced (*p* > 0.05) by the addition of different fat sources in the diet. 

The animals in this study spent 52.5% of the day idling (12.6 h), 34.8% of the day ruminating (8.4 h), and 12.7% of the day feeding (3.0 h). On the other hand, the rumination efficiencies of DM (*p* < 0.001) and NDF (*p* = 0.003) were higher in lambs fed the CSFA diet. No differences were recorded (*p* > 0.05) in the number of cuds per day or the DM for cud (Table 2).

### 3.2. Carcass Traits and Meat Quality

The inclusion of different fat sources did not influence (*p* > 0.05) the morphometric measurements of the lambs except in the estimation of the carcass compactness index (CCI), where the CSFA diet promoted (*p* = 0.001) a higher CCI in feedlot lambs (Table 3).

The addition of different fat sources in the diet did not influence (*p* > 0.05) the CL, subcutaneous fat thickness, kidney fat, and marbling of feedlot lambs. The CSFA diet provided higher weights (*p* < 0.05) and performance in the lamb carcass compared to lambs fed with other fat sources (Table 4).

The physicochemical characteristics and centesimal composition of the LL muscle were not influenced (*p* > 0.05) by the inclusion of different fat sources in the diet of feedlot lambs (Table 5).

### 3.3. Fatty Acids

The SFA C12:0, C14:0, C15:0, and C16:0 and UFA C16:1 *c9* and C18:2 *t10c12* in the LL muscle of lambs fed different fat sources were similar (*p* > 0.05). However, stearic acid (C18:0) was 49.6% higher in the CG diet compared to that in the CSFA diet, which in turn had a lower content of this fatty acid. The highest concentration of branched-chain fatty acid (BCFA) C17:0 was in the NAF diet, and that of C18:0 was in the CSFA diet (Table 6).

The LL muscle of the lambs fed the CSFA, SO, and CG diets showed the highest concentrations of CLA (C18:2 *c9t11*) and the lowest concentrations of oleic acid (C18:1 *c9*). The content of vaccenic acid (C18:1 *t11*) was higher (*p* < 0.001) in lambs fed the CSFA diet, 324% higher than the concentration observed in the NAF diet. The production of FA derived from biohydrogenation was higher for the CSFA diet. The polyunsaturated fatty acids C18:2 *c9c12*, C18:3 n-3, C20:4 n-6, C20:5 n-3, C22:5 n-3, and C22:6 n-3 were always higher (*p* < 0.05) in the LL muscle from lambs fed the WSB diet (Table 6).

While the WSB diet promoted the highest concentration (*p* < 0.05) of total saturated fatty acids (ΣSFA), the CSFA and SO diets showed the lowest (*p* = 0.025) (Table 7). There were no changes (*p* > 0.05) in the concentrations of total monounsaturated fatty acids (ΣMUFA). However, the addition of different sources of fats in the feeding of feedlot lambs promoted a higher concentration (*p* < 0.001) of total polyunsaturated fatty acids (ΣPUFA) in meat compared to the NAF diet. 

The PUFA:SFA ratio was higher (*p* < 0.001) in the CSFA and SO diets. The inclusion of whole soybeans, soybean oil, and corn germ promoted an increase (*p* < 0.05) in the concentration of fatty acids in the omega 6 series (ΣPUFA n-6). However, the concentration of fatty acids in the omega 3 series (ΣPUFA n-3) was higher (*p* < 0.001) with the addition of whole soybeans in the diet. The highest n-6:n-3 ratio was observed with the CG diet, and the lowest was observed with the WSB (*p* < 0.001). The WSB diet promoted (*p* < 0.01) the highest atherogenic (AI) and thrombogenic (TI) indexes. The hypocholesterolemic and hypercholesterolemic ratio (h:H) increased (*p* = 0.006) with the NAF, SO, and CG diets (Table 7).

A higher activity (*p* < 0.05) of the enzyme Δ9 C18 desaturase was found in the meat of lambs fed the NAF diet. On the other hand, the highest activity (*p* < 0.001) of elongase was estimated for the SO and GC diets (Table 7).

## 4. Discussion

### 4.1. Feeding Behaviour

The higher total intake of DM and NDF from the NAF and CSFA diets suggests that chemically protected fats avoid adverse effects on the ruminal microbiota, which did not occur in the WSB, SO, and CG diets. In these latter diets, EE may have contributed to reducing intake [30], promoting two apparent effects: selectivity and/or toxicity. However, the intake changes did not compromise the time spent on the main activities of feeding behavior (ruminating, feeding, idling, and chewing).

According to Owens and Basalam [31], feeding time can vary from 1 to 12 h/day. The average feeding time of 3 h/day in the present experiment is probably related to the type of diet (total mixed ration, 60% concentrate), thus avoiding the animals spending excessive time in the trough with the selection of specific ingredients.

In the current study, the concentrations of fibrous carbohydrates in the diets (NDF and ADF) were similar, showing an average rumination time of 8.4 h/day. Costa et al. [32] indicated that, regardless of the concentration of nondigestible neutral detergent fiber in the diet, lambs with high intake rates have shorter rumination times. Numerically, the rumination time was short in lambs fed the CSFA diet, which may have promoted higher DM and NDF rumination efficiencies.

### 4.2. Carcass Traits and Meat Quality

The absence of a significant effect in the morphometric measurements of the carcass (except for CCI) can be explained by the isometric development and parallel evolution of the carcass growth, which resulted in an anatomical harmony [33]. On the other hand, the low standard error of the mean suggest that the effect of environmental factors was not significant [34].

The use of lipids in diets for ruminants increases the content of ME as well as protein use efficiency [35]. The addition of fatty acid salts promotes higher DM intake [36]. Therefore, the higher carcass yields observed in lambs fed the CSFA diet can be explained due to the better use of DM for growth development.

Medium and long-chain fatty acids generate ATP more efficiently than volatile fatty acids generated in the rumen [1]. In this way, this energy contributed to muscle formation and growth in the animals of the present study. Bhatt et al. [37] observed a higher performance of lambs fed diets with high levels of PUFAs.

The CSFA diet provided higher productive performance. This result is confirmed by the compactness carcass index, which indicates a higher deposition of muscle tissue per unit of length. The productive performance is related to the process of synthesis, degradation, and deposition of body proteins, which needs higher amounts of energy that can be supplied by diet [38].

Nutritional management is one of the main factors that affect the quantity and quality characteristics of the carcass and meat [39]. In this study, no influence of the inclusion of fat sources in the diets was observed on the physicochemical characteristics of the carcass or centesimal composition of the LL muscle, which is in agreement with several studies in this area [40,41,42].

The pH of sheep meat is close to neutral in live animals and decreases to 5.4–5.8 after 24 h of slaughter under normal conditions [43,44,45]. In the current study, the mean pH value of the meat was 5.8. The result obtained suggests that the animals did not suffer stress before slaughter or increased glycolysis after slaughter [46]. The importance of evaluating the meat pH lies in its importance in influencing other parameters such as color, tenderness, and microbiological stability, among others [44,46].

The average CL value of 31% was lower than the values (35.11–38.4%) reported in lamb meat [45,47,48]. The CL is correlated with the amount of intra-, inter-, and extra-muscular lipids. Therefore, the similarity between the carcasses can be supported by the lack of differences in their lipid content (subcutaneous and marbling fat). It is important to highlight that, the lower the CL values, the better the benefits concerning the tenderness of the meat obtained [49].

The SF is related to factors such as age [50], collagen solubility [51], lipid accumulation [49], and weight of the animals. Values between 2.35 and 4.08 kgf/cm^2^ can be observed in sheep meat [52], considering ideal values at less than 3.4 kgf/cm^2^ [53]. Therefore, the FS of 2.8 kgf/cm^2^ observed in the current study is considered soft.

Although the EE content of diets with some fat sources (6.31%) was almost double that of the NAF diet (3.26%), the proximal composition of the LL muscle was similar among lambs. Renal, subcutaneous, and marbling fat values in lamb carcasses indicate that dietary lipids were primarily used as an energy source for production and were stored in smaller amounts [54].

### 4.3. Fatty Acid Profile

Odd- and branched-chain fatty acids (OBCFA) are formed by ruminal bacteria from the elongation of propionate, valerate, valine, leucine, and isoleucine [55]. Animals fed a higher amount of NFC have a higher proportion of OBCFA precursors [56]. Dietary fat inclusion (WSB, CSFA, SO, and CG) were related to decreased levels of NFC in the diets, which explains the reason for the concentrations of FA C17:0 *iso* being higher with the NAF diet.

The C18:0 *iso* concentration was higher with the CSFA diet, and this can be explained by the fact that calcium salts do not affect the ruminal cellulolytic bacterial population [9]. Related to this, even- and branched-chain fatty acids, mainly *iso*, are correlated with a higher population of cellulolytic bacteria in the rumen (*R. albus, B. fibrisolvens, and R. flavefaciens*) [57].

The higher content of C18:2 *c9c12* in the LL muscle was observed in lambs fed the WSB and CG diets. This result suggests a natural protection of fatty acids against the biohydrogenation process [58]. Soybean oil has no protection against the enzymatic action of biohydrogenation; consequently, the concentration of C18:2 *c9c12* in the LL muscle of lambs fed this diet was lower.

The level of dissociation of calcium salts of fatty acids is higher as the concentration of unsaturated fatty acids increases [59], which explains the lower concentration of C18:2 *c9c12* in the LL muscle of lambs fed the CSFA diet.

The highest concentrations of C18:2 *c9t11* were observed in the LL muscle of lambs fed the CSFA, SO, and CG diets. This result indicates that lipid biohydrogenation occurred in these diets. According to Fruet et al. [60], the principal conjugated linoleic acid (CLA) generated in the biohydrogenation process is C18: 2 *c9t11* (58.9%).

The higher concentration of C18:1 *t11* observed in the LL muscle of lambs fed the CSFA diet suggest an accumulation of this fatty acid. The increase of C18:1 *t11* inhibits the final step of biohydrogenation [11]. In this way, this diet has a higher amount of C18:1 *t11* and a lower concentration of C18:0, which indicates incomplete biohydrogenation. On the other hand, the higher concentration of C18:0 found in the LL muscle of lambs fed the CG diet suggests complete biohydrogenation of the MUFA and PUFA of this diet.

The high concentrations of C18:3 n-3 and C18:2 n-6 in the LL muscle of lambs fed the WSB diet suggest a lower biohydrogenation of this diet, which explains the higher total concentration of SFA. Fatty acids were protected by cotyledons and other grain seed structures [9], avoiding ruminal bacteria activity and reaching the duodenum in almost the same proportion in which it was supplied [55].

The n-6 (20:4 n-6) and n-3 (C20:5 n-3, C22:5 n-3, and C22:6 n-3) fatty acids found in the LL muscle may come from diet or elongation of the 18:2 n-6 and C18:3 n-3 fatty acids [61]. It should be noted that the inclusion of different fat sources in the diet of lambs, regardless of the degree of ruminal biohydrogenation, increased the PUFA profile in meat [62].

The LL muscle of lambs fed the WSB diet showed higher concentrations of n-6 and n-3 fatty acids, related to the degree of protection of long-chain fatty acids [55] with this diet. This result suggests that whole soybean promoted a higher protection of fatty acids from biohydrogenation, resulting in a higher concentration of PUFA in meat. On the other hand, the PUFA synthesis in these animals is lower due to the lower activities of elongase and desaturase (Δ^5^ and Δ^6^) enzymes [63].

The SO and CG diets promoted higher concentrations of n-6 fatty acids and low n-3 fatty acids, indicating greater biohydrogenation of n-3 fatty acids, confirmed by high levels of C18:2 *c9t11*. The PUFA concentrations are the result of the elongation of fatty acids due to the high elongase activity with these diets.

Stearic acid (C18:0) can be converted to oleic acid (C18:1 *c9*) through the enzymatic activity of ∆^9^ desaturase, through a dehydrogenation reaction, and through the addition of a stereospecific double bond within the chain [63]. The ∆^9^ C18 desaturase activity observed in the meat of lambs fed the NAF diet explains the higher concentration of C18:1 *c9* in the LL muscle of these animals.

The n-6/n-3 ratio is related to the increase in the levels of cholesterol in the blood. In this way, the recommended value for the prevention of cardiovascular diseases should be less than 4 [64]. All treatments showed values within the recommended range. However, the highest value obtained with the CG diet was influenced by the concentrations of n-3 fatty acids.

AI and TI show the trend in the formation of fatty plaques in the arteries that promote cardiovascular diseases [54,64]. The recommended values for AI and TI should be less than 1.0 and 0.5, respectively [65]. Since AI and TI are functions of the sum of the fatty acids UFA, MUFA, n-6, and n-3, the high values of these free acids influenced the higher values of AI and TI in the LL muscle of lambs fed the WSB diet.

The AI in the LL muscle of the lambs was less than 1.0, and the TI was higher than 0.5. However, the AI and TI values were less than 1.0 and 2.0, respectively. The values observed in the current study are similar to those reported by Bhatt et al. [54] in lambs fed with calcium salts of fatty acids and flaxseed.

The h:H rate below 2.0 is ideal for positively modulating the cholesterol transport mechanism by lipoproteins and the prevention of cardiovascular diseases [64]. The C14:0 and C16:0 values were similar between diets, with the highest concentrations observed in lambs fed the NAF, SO, and CG diets due to the concentrations of the C18:1 *c9*, n-6, and n-3 fatty acids.

## 5. Conclusions

The inclusion of 3.5% of calcium salts of fatty acids as a source of protected fat in the total diet of feedlot lambs results in higher slaughter weight, carcass weight and yield, conformation, finishing, compactness index, and loin eye area of the carcass.

Calcium salts of fatty acids in diets also improve the profile of fatty acids in meat, especially those that are bioactive and important for human health, such as intermediate fatty acids from biohydrogenation and PUFA; in addition, they reduce the SFA content.

## Figures and Tables

**Table 1:** Proportion of ingredients, chemical composition, and fatty acid profile of the experimental diets.

Item	Experimental Diets				
	NAF	WSB	CSFA	SO	CG
Ingredients (g/kg DM)					
Tifton-85 hay	400.0	400.0	400.0	400.0	400.0
Ground corn	459.0	353.0	414.0	419.0	364.0
Soybean meal	120.0	30.0	130.0	130.0	115.0
Soybean oil	-	-	-	30.0	-
Whole soybean	-	200.0	-	-	-
Calcium salt of fatty acids	-	-	35.0	-	-
Corn germ	-	-	-	-	100.0
Urea	6.0	2.0	6.0	6.0	6.0
Mineral supplement ^1^	15.0	15.0	15.0	15.0	15.0
Chemical composition (g/kg DM)					
Dry matter (g/kg as fed)	900.5	903.0	903.6	903.8	904.6
Ash	52.5	54.8	57.3	52.6	52.2
Crude protein	175.3	187.4	176.2	176.7	177.2
Ether extract	32.6	61.6	61.2	61.1	68.5
Neutral detergent fiber ap ^2^	349.5	358.2	345.9	346.5	365.9
Acid detergent fiber ap ^3^	171.9	178.1	171.4	171.5	191.2
Lignin	35.1	35.0	34.6	34.7	37.7
Non-fibrous carbohydrates	390.0	338.0	359.4	363.1	336.2
Total digestible nutrients	708.4	740.6	742.3	746.7	737.9
Neutral detergent insoluble protein	30.7	41.0	30.3	30.4	34.1
Acid detergent insoluble protein	10.3	13.6	10.1	10.1	10.4
Fatty acid profile (mg/100 g)					
Caprylic (C8:0)	1.0	1.0	1.0	1.3	1.3
Capric (C10:0)	5.1	5.1	4.7	5.3	6.8
Lauric (C12:0)	10.2	10.1	9.6	10.7	12.0
Myristic (C14:0)	61.2	60.2	56.7	61.7	113.0
Palmitic (C16:0)	476.7	497.0	530.7	620.8	987.3
Palmitoleic (C16:1)	13.0	13.2	14.0	14.3	20.2
Stearic (C18:0)	112.4	122.2	149.4	154.9	210.1
Oleic (C18:1 n-9)	786.8	804.7	967.0	916.5	2146.2
Linoleic (C18:2 n-6)	1250.5	1356.5	1655.3	1465.5	2949.8
α-linolenic (C18:3 n-3)	57.9	77.9	112.2	78.2	85.9

^1^ Provides per kg of active element: calcium—240.00 g, phosphorus—71.00 g, potassium—28.20 g, sulfur—20.00 g, magnesium—20.00 g, copper—400.00 mg, cobalt—30.00 mg, chromium—10.00 mg, iron—250.00 mg, iodine—40.00 mg, manganese—1350.00 mg, selenium—15.00 mg, zinc—1700.00 mg, and fluorine (max.)—710.00mg; ^2^ neutral detergent fiber corrected for ash and protein; ^3^ acid detergent fiber corrected for ash and protein.

**Table 2:** Feeding behavior of feedlot lambs fed diets containing different fat sources.

Item	Experimental Diets					SEM	*p*-Value
	NAF	WSB	CSFA	SO	CG		
Total Intake, kg							
Dry matter	115.9a	95.3b	114.0a	100.8b	90.6b	2.455	<0.001
Neutral detergent fiber	44.7a	38.6b	43.5a	38.5b	36.8b	0.879	0.010
Time, h/day							
Rumination	8.6	8.4	7.7	8.8	8.4	0.145	0.197
Feeding	3.2	2.9	3.1	2.6	3.4	0.106	0.081
Idling	12.2	12.7	13.2	12.6	12.2	0.196	0.476
Chewing	11.8	11.3	10.8	11.4	11.8	0.196	0.476
Efficiency, g DM/h							
Feeding	372.5	369.2	402.3	437.9	304.5	14.851	0.057
Rumination	138.2ab	122.1b	159.9a	122.1b	115.5b	3.779	<0.001
Efficiency, g NDF/h							
Feeding	134.2	135.7	139.6	153.5	112.1	5.208	0.157
Rumination	49.9ab	45.0b	55.6a	42.7b	42.5b	1.333	0.003
Cuds							
Cuds, n°/day	705.4	691.9	723.0	650.2	658.5	14.393	0.467
Dry matter for cud, g	1.7	1.5	1.8	1.6	1.5	0.049	0.309

Different lowercase letters indicate differences between columns. Diets: NAF, the basal diet without the addition of a fat source; WSB, whole soybean; CSFA, calcium salt of fatty acids; SO, soybean oil; CG, corn germ.

**Table 3:** Morphometric measurements (cm) of the carcass of feedlot lambs fed diets containing different fat sources.

Item	Experimental Diets					SEM	*p*-Value
	NAF	WSB	CSFA	SO	CG		
External length	55.0	55.0	57.1	56.5	55.0	0.460	0.429
Internal length	53.9	53.9	54.9	54.4	52.9	0.342	0.427
Leg length	38.7	37.9	37.7	39.2	38.1	0.339	0.626
Leg circumference	44.9	42.2	45.9	43.6	42.3	0.503	0.066
Thoracic width	26.0	25.2	27.1	25.9	25.3	0.295	0.221
Thoracic depth	25.9	25.0	26.8	25.8	25.1	0.252	0.177
Thoracic perimeter	78.4	75.3	81.0	74.6	75.3	0.834	0.062
Rump width	21.6	20.8	23.0	21.2	21.1	0.319	0.193
Rump perimeter	53.7	50.8	56.3	52.3	52.6	0.765	0.207
Carcass compactness index	0.35ab	0.31b	0.38a	0.33ab	0.30b	0.008	0.001
Leg compactness index	0.56	0.55	0.61	0.54	0.56	0.011	0.222

Different lowercase letters indicate differences between columns. Diets: NAF, the basal diet without the addition of a fat source; WSB, whole soybean; CSFA, calcium salt of fatty acids; SO, soybean oil; CG, corn germ.

**Table 4:** Measures of yield and carcass quality of feedlot lambs fed diets containing different fat sources.

Item	Experimental Diets					SEM	*p*-Value
	NAF	WSB	CSFA	SO	CG		
Carcass traits							
Slaughter body weight, kg	42.8ab	39.1b	45.5a	42.3ab	38.8b	0.700	0.006
Hot carcass weight, kg	19.2ab	16.5b	21.2a	18.1ab	16.2b	0.466	0.001
Cold carcass weight, kg	19.1ab	16.4b	21.0a	18.0ab	16.1b	0.461	0.001
Cooling losses, kg	0.493	0.235	0.645	0.364	0.686	0.078	0.318
Hot carcass yield, %	44.6ab	42.1b	46.5a	42.8ab	41.5b	0.500	0.004
Cold carcass yield, %	44.4ab	42.0b	46.2a	42.7ab	41.2b	0.497	0.006
Subjective evaluation							
Conformation	3.4ab	3.3ab	3.5a	3.1b	3.2b	0.046	0.020
Finishing	3.2ab	3.0ab	3.4a	3.0ab	2.9b	0.053	0.019
Marbling	2.7	2.5	2.8	2.1	2.0	0.109	0.086
Kidney Fat	2.2	2.2	2.3	2.2	2.1	0.057	0.975
*Longissimus lumborum* muscle							
Loin, kg	0.96ab	0.84b	1.14a	0.97ab	0.80b	0.030	0.001
Loin eye area, cm^2^	12.3ab	11.1b	14.0a	12.0ab	10.0b	0.368	0.004
Subc. fat thickness, mm	2.6	2.1	2.6	2.9	2.8	0.190	0.708

Different lowercase letters indicate differences between columns. Diets: NAF, the basal diet without the addition of a fat source; WSB, whole soybean; CSFA, calcium salt of fatty acids; SO, soybean oil; CG, corn germ.

**Table 5:** Physicochemical characteristics and centesimal composition of the longissimus lumborum (LL) muscle of lambs fed diets containing different fat sources.

Item	Experimental Diets					SEM	*p*-Value
	NAF	WSB	CSFA	SO	CG		
Physicochemical characteristics							
pH 45 min	7.1	7.0	7.1	7.1	7.1	0.040	0.902
pH 24 h	5.9	5.6	5.9	5.7	5.7	0.035	0.149
T° 45 min, °C	31.2	31.9	30.9	30.9	31.2	0.294	0.813
T° 24 h, °C	9.6	9.2	9.4	9.6	9.5	0.103	0.679
Cooking losses, %	29.9	25.5	39.1	36.4	25.1	2.103	0.106
Shear force, kgf/cm^2^	2.7	2.8	2.9	2.7	2.7	0.069	0.933
Lightness, L *	40.2	40.8	38.6	40.4	41.6	0.489	0.401
Redness, a *	21.7	22.0	22.5	22.7	22.5	0.201	0.572
Yellowness, b *	6.9	7.6	7.3	7.8	7.8	0.245	0.757
Centesimal composition of LL muscle (g/kg)							
Dry matter	273.6	277.8	269.6	268.7	266.4	0.176	0.253
Moisture	726.4	722.2	730.4	731.3	733.6	0.176	0.253
Ash	8.6	9.0	8.7	9.2	9.3	0.017	0.679
Ether extract	37.3	39.6	36.5	30.2	31.8	0.157	0.277
Crude protein	213.7	214.9	217.2	221.3	229.5	0.207	0.096

Different lowercase letters indicate differences between columns. Diets: NAF, the basal diet without the addition of a fat source; WSB, whole soybean; CSFA, calcium salt of fatty acids; SO, soybean oil; CG, corn germ.

**Table 6:** Fatty acid composition (mg/100 g of meat) of the LL muscle of lambs fed diets containing different fat sources.

Fatty Acid	Experimental Diets					SEM	*p*-Value
	NAF	WSB	CSFA	SO	CG		
Saturated fatty acids							
Lauric (C12:0)	3.0	3.14	2.6	2.2	2.7	0.147	0.271
Myristic (C14:0)	76.5	73.95	72.6	59.7	65.5	3.141	0.444
Pentadecanoic (C15:0)	10.7	12.06	8.5	9.0	10.6	0.562	0.241
Palmitic (C16:0)	911.5	863.1	779.4	698.7	767.9	31.256	0.242
Stearic (C18:0)	581.1b	735.5ab	538.6c	609.4bc	805.5a	32.856	0.028
Branched-chain fatty acids							
C14:0 *iso*	1.2	1.59	1.0	1.1	1.5	0.098	0.232
C15:0 *iso*	4.9	5.60	3.4	4.1	4.6	0.304	0.199
C15:0 *anteiso*	5.0	6.4	3.8	4.9	5.8	0.353	0.206
C16:0 *iso*	6.4	6.8	4.4	5.9	6.3	0.400	0.400
C17:0 *iso*	1.4a	0.8b	0.8b	0.6b	0.5b	0.086	0.008
C18:0 *iso*	1.5c	1.3c	3.2a	2.2b	1.8bc	0.160	<0.001
Monounsaturated fatty acids							
Palmitoleic (C16:1)	55.7	55.2	51.8	44.9	39.7	2.247	0.089
Oleic (C18:1 n-9)	1741.4a	1483.8ab	1215.0b	1304.8b	1360.8b	58.839	0.041
Vaccenic (C18:1 *t11*)	33.8d	62.8cd	143.1a	101.9b	88.4bc	8.826	<0.001
Polyunsaturated fatty acids							
Linoleic (C18:2 *c9c12)*	103.2c	199.4a	158.6b	159.8b	170.3a	8.609	0.001
CLA ^1^	7.5b	9.7b	22.5a	18.3a	19.0a	10.439	0.002
CLA isomer (C18:2 *t10c12)*	0.9	0.7	1.2	0.9	0.7	0.075	0.149
α-linolenic (C18:3 n-3)	6.9c	12.1a	7.9bc	9.2b	5.2d	0.543	<0.001
Arachidonic (C20:4 n-6)	36.8b	42.5a	29.7c	42.8a	42.4a	1.280	<0.001
Eicosapentaenoic (EPA; C20:5 n-3)	4.3a	4.9a	3.1b	4.6a	3.0b	0.198	<0.001
Docosapentaenoic (DPA; C22:5 n-3)	6.3bc	8.3a	5.3c	7.1ab	5.7bc	0.307	0.004
Docosahexaenoic (DHA; C22:6 n-3)	1.2ab	1.9a	0.8bc	1.4ab	1.1bc	0.113	0.037

Different lowercase letters indicate differences between columns. Diets: NAF, the vasal diet without the addition of a fat source; WSB, whole soybean; CSFA, calcium salt of fatty acids; SO, soybean oil; CG, corn germ; ^1^ CLA, conjugated linoleic acid (coeluted peak with C18:2 t7c9 and C18:2 c9t11).

**Table 7:** Fatty acid classes (mg/100 g of meat), ratios, indexes, and enzyme activity in the LL muscle of lambs fed diets containing different fat sources.

Item ^1^	Experimental Diets					SEM	*p*-Value
	NAF	WSB	CSFA	SO	CG		
Σ SFA	1635.1ab	1962.1a	1437.4b	1417.8b	1695.9ab	58.864	0.025
Σ MUFA	1877.2	1754.7	1448.3	1478.7	1513.1	57.893	0.065
Σ PUFA	178.8b	270.0a	247.8a	254.4a	250.5a	7.882	<0.001
PUFA:SFA	0.11c	0.14b	0.17a	0.18a	0.15b	0.006	<0.001
Σ PUFA n-6	39.1b	45.4a	32.0c	46.0a	45.2a	1.355	<0.001
Σ PUFA n-3	18.3bc	25.4a	16.4c	21.1b	15.6c	0.847	<0.001
n-6:n-3	2.07b	1.61c	1.98b	1.96bc	3.33a	0.130	<0.001
AI	0.60b	0.67a	0.62ab	0.53c	0.57bc	0.013	0.004
TI	1.7b	2.0a	1.8b	1.7b	2.0a	0.040	<0.001
h:H	1.8a	1.6b	1.5b	1.8a	1.8a	0.036	0.006
Δ^9^ C18	74.4a	65.6cd	69.3b	68.1bc	63.6d	0.892	<0.001
Elongase	70.5b	70.6b	67.9c	72.7a	72.8a	0.465	<0.001
DFA	2631.6	2869.9	2234.8	2342.6	2646.6	81.703	0.097

Different lowercase letters indicate differences between columns. Diets: NAF, the basal diet without the addition of a fat source; WSB, whole soybean; CSFA, calcium salt of fatty acids; SO, soybean oil; CG, corn germ. ^1^ AI, atherogenicity index; TI, thrombogenicity index; h:H, hypocholesterolemic–hypercholesterolemic ratio; Δ^9^ C16, delta-9 hexadecanoic-CoA desaturase; Δ^9^ C18, delta-9 octadecanoic-CoA desaturase; DFA, desirable fatty acids.

## Data Availability

No new data were created or analyzed in this study. Data sharing is not applicable to this article.
